# Reproductive health characteristics among women living in severe poverty in urban Haiti

**DOI:** 10.1186/s12978-025-02219-3

**Published:** 2025-11-24

**Authors:** Wheytnie Alexandre, Anju Ogyu, Rodney Sufra, Lily D. Yan, Marie M. Deschamps, Catherine Bennett, Jean William Pape, Laura C. Alonso, Vanessa Rouzier, Margaret L. McNairy

**Affiliations:** 1 Center for Global Health, Weill Cornell Medicine, NYC, NY USA; 2 Division of General Internal Medicine, Weill Cornell Medicine, NYC, NY USA; 3 Haitian Group for the Study of Kaposi’s Sarcoma and Opportunistic Infections (GHESKIO), Port-au-Prince, Haiti; 4 Division of Endocrinology, Diabetes & Metabolism, Weill Cornell Medicine, NYC, NY USA

**Keywords:** Reproductive health, Adverse pregnancy outcomes, Haiti, Pregnancy loss, Miscarriage, Stillbirths, Pregnancy complications

## Abstract

**Background:**

Data on women’s reproductive health in settings facing extreme poverty are limited. We describe the reproductive health characteristics across the life course of women living in urban Port-au-Prince, Haiti, and identify factors associated with adverse pregnancy outcomes (APO).

**Methods:**

Data were sourced from the Haiti Cardiovascular Disease Cohort Study, a population-based observational study in Port-au-Prince. This analysis includes all women who completed a reproductive health questionnaire, which included self-reported age of menarche, menopause, infertility, menstruation abnormalities, and APO, defined as a history of pregnancy loss, preterm births, and pregnancy complications. We performed univariable and multivariable log-binomial regression to identify factors associated with APO.

**Results:**

Among 1746 women in the parent cohort, 1,163 (66.6%) women reported reproductive health data. The median age was 43 years (IQR 31–55). The median age at menarche was 14 years (IQR 12–16). A downward trend in the age of menarche was observed over time: women born from 1930 to 1970 reported a median age of 15 years, compared to 13 years among women born from 1990 to 2012 (*p* < 0.001). 11% (11.0%; *n* = 130) of the participants reported a history of infertility. The median age of reported menopause was 48 years (IQR, 44–50; *n* = 540), with approximately 37.4% (*n* = 203) experiencing menopause before the age of 45. Among 1007 women with *≥* one pregnancy, 61.7% (*n* = 623) experienced an APO, and 20.3% (*n* = 206) reported two or more APO. The most common APO was pregnancy loss (*n* = 515, 51.0%), pregnancy complications (*n* = 244, 24.0%), and preterm births (*n* = 132, 13.0%). Factors associated with a history of APO included hypertension, body mass index > 25 kg/m2, higher education, and a moderate perceived stress score.

**Conclusion:**

Among women living in extreme poverty in Haiti, we found a high prevalence of adverse reproductive health outcomes, namely APO, and a temporal trend of a decreasing age of menarche across time. Adverse reproductive health outcomes have been associated with increased chronic disease, and additional research is needed to establish if these factors are associated with increased cardiometabolic disease in extremely poor settings to identify targets for future prevention and treatment.

**Supplementary Information:**

The online version contains supplementary material available at 10.1186/s12978-025-02219-3.

## Abstract (plain language summary)

Data on women’s reproductive healthcare are not readily available in settings of extreme poverty, including Haiti; yet they are essential for improving women’s health in these contexts.

We report data from women participants in the population-based Haiti Cardiovascular Disease Cohort Study. We used regression analysis to identify factors associated with adverse pregnancy outcomes.

Among 1163 women, the median age was 43 years. The majority (68%, *n* = 791) earned less than 1 USD, and 46% (*n* = 530) had only a primary-level education. We noted a statistically significant decline in the age at which women experience their first menstrual period, from 15 years for those born between 1930 and 1970 to 13 years among those born between 1990 and 2012. The median age at menopause was 48 years (*n* = 540). Among women who had one or more pregnancies, the vast majority, 61.7% (*n* = 623), reported experiencing adverse pregnancy outcomes. Factors associated with these outcomes included hypertension, obesity, and stress.

Among women living in extreme poverty in urban Haiti, we found that two-thirds experienced one or more adverse pregnancy outcomes. Adverse pregnancy outcomes have been linked to chronic diseases like heart disease and may help identify women at high risk in low-resource settings, allowing for targeted prevention and treatment.

**Supplementary Information:**

The online version contains supplementary material available at 10.1186/s12978-025-02219-3.

## Introduction

Women’s reproductive health gained international recognition as a valuable metric of development at the 1994 United Nations (UN) International Conference on Population and Development (ICPD) [1, 2]. Reproductive health data previously focused on family planning and sexual health, as related to prevention of infectious diseases, primarily HIV/AIDS and other sexually transmitted infections [3, 4], and more recently broadened to include age at menarche, infertility and related disorders, contraception, parity, adverse pregnancy outcomes (APO), and menopause, due to their association with maternal mortality, chronic diseases, and cancer risks [5, 6]. Local data to describe the reproductive health profile of women living in the world’s poorest places, including slum neighborhoods, is scarce but needed to guide women’s health programs to achieve the 2030 UN Sustainable Development Goal of integration of reproductive health into national strategies to improve women’s health [5, 7].

This sub-study aims to describe the type and magnitude of reproductive health characteristics and factors associated with adverse pregnancy outcomes among women enrolled in the Haiti Cardiovascular Disease Cohort Study. We hypothesize that the prevalence of adverse pregnancy outcomes is high and is associated with low socioeconomic status (low income, low education), and with traditional cardiovascular risk factors, primarily obesity and hypertension. Understanding the critical milestones of our cohort’s reproductive life cycle—such as the age of menarche, menstrual abnormalities, history of infertility, parity history, APO, and menopausal history can help identify targets to improve reproductive health [5].

## Methods

### Cohort description

This study uses enrollment data from the Haiti Cardiovascular Disease Cohort Study (clinicaltrials.gov NCT03892265). The Haiti Cardiovascular Disease Cohort is a longitudinal observational study involving 3,005 adults aged 18 years and older, recruited through multistage random sampling of national census blocks between March 2019 and August 2021 at the Haitian Group for the Study of Kaposi Sarcoma (GHESKIO) clinic in Port-au-Prince, Haiti. The methodology and detailed research protocol have been published previously [8]. The sampling strategy was based on census blocks from the Institut Haitien de Statistique et d’Informatique, using ArcGIS Software to randomly assign waypoints to census blocks in proportion to their population size, and then locating the nearest household to these waypoints for study recruitment. Inclusion criteria for the original cohort included individuals aged ≥ 18 years and residing in Port-au-Prince. Participants with significant medical illnesses leading to a life expectancy of less than one year or with significant cognitive impairment were excluded. For this sub-study, women participants were asked to complete an interviewer-administered reproductive health survey, which was added to the longitudinal study on August 1, 2023, and administered at the annual study visit. A research nurse administered the survey.

### Sample size

**Fig. 1:**
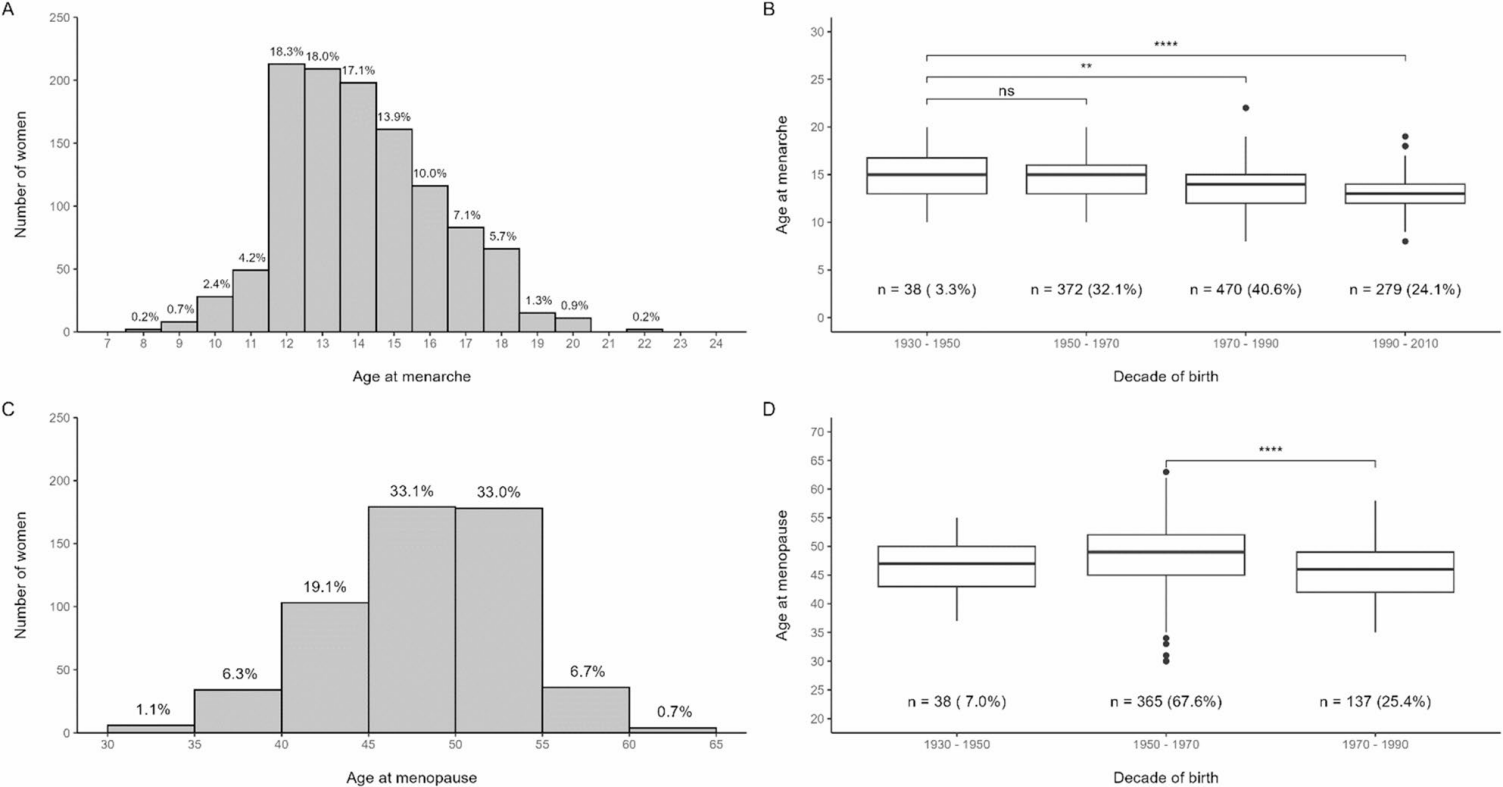
Age of distribution of menarche and menopause in the Haiti CVD Cohort. **A **Distribution of reported ages of menarche.** B **Trends in the reported age of menarche across decades of birth.** C **Distribution of reported ages at menopause.** D **Trends in the reported age of menopause across decades of birth

A total of 1,746 women participants were enrolled in the Haiti Cardiovascular Disease Cohort Study, and 1,163 (66.6%) completed the reproductive health questionnaire between August 11, 2023, and December 26, 2024, which marks the end of data collection for this analysis. Reasons for not completing the questionnaire included 46 (2.6%) deaths, 10 (0.5%) who had exited the study, and 527 (30.0%) who did not attend a clinic-based visit during this period (Fig. 1, supplementary file).

### Data collection and measures

At study enrollment, measures included age, sex, sociodemographic factors (income, education, marital status, and residence), clinical measures (height, weight, blood pressure (BP)), and past medical history. Health behavior measures include smoking status, alcohol use, diet, and physical inactivity, as assessed using WHO STEPS survey questionnaires [9]. Depression categories were measured by the Patient Health Questionnaire translated into Haitian-Creole (PHQ-9) [10]. The Haitian-Creole version of the PHQ-9 has been shown to be an adequate tool to measure depression in Haiti in prior research [11]. Moderate to severe levels of depression were combined, given the low prevalence of participants with severe depression. Stress was stratified using the Perceived Stress Scale translated into Haitian-Creole and categorized into low, moderate, and high levels [12]. Hypertension was defined per WHO criteria as systolic blood pressure ≥ 140 mmHg or diastolic blood pressure ≥ 90 mmHg or taking blood pressure medications [13].

The reproductive health questionnaire included questions adapted from the National Health and Nutrition Examination Survey (NHANES) on reproductive health surveys conducted by the Centers for Disease Control and Prevention [14]. Questions include participants’ age at menarche, age at menopause, menstrual patterns, history of infertility and oligomenorrhea, parity, pregnancy outcomes, and breastfeeding history. Questions are detailed in Supplement Table 2. Based on prior literature, the age of menarche was categorized as early if it occurs before age 12 and as late if it occurs after age 15 [15]. Infertility is determined by asking, “Have you had difficulty getting pregnant in the past or now,” and does not specify 12 months or longer, as recommended by the International Federation of Gynecology and Obstetrics. (FIGO) [16]. A history of pregnancy loss was determined as the difference between total pregnancies and live births. A history of pregnancy loss was assigned if this value was ≥ 1. Pregnancy loss encompasses stillbirths and miscarriages. We are unable to determine the specific timing of the pregnancy losses and, therefore, could not distinguish between stillbirths and miscarriages in our analysis. Adverse pregnancy outcomes (APO) were defined as a history of pregnancy loss, preterm births, and pregnancy complications, which included hypertensive disorders of pregnancy, gestational diabetes, and small size for gestational age. Supplementary Table 3 summarizes the operational definitions for all reproductive health variables, the study’s primary outcomes.

### Statistical analysis

Deidentified data were abstracted from the NIH REDCap database of the Haiti CVD Cohort Study. Reproductive characteristics were described using summary statistics (medians and interquartile ranges for continuous variables, and counts and proportions for categorical variables), as well as histograms and box plots, to identify outliers and data trends. For menopause, extreme outliers (age of menopause < 30) were excluded. Missing data are reported for each reproductive health variable. We evaluate the age of menarche and menopause by estimating the year of birth using the age at the enrollment visit. Birth years were categorized by two decades (1930–1950, 1950–1970, 1970–1990, and 1990–2010). Differences in age of menarche and menopause across categories of birth years were assessed using the Kruskal-Wallis rank sum test. Where significant, post-hoc pairwise comparisons were conducted using the Wilcoxon rank sum test. Log-binomial regression was used to estimate the prevalence ratios (PR) for the association between risk factors and APO, given the high prevalence of APO, fitting both univariable and multivariable regression. For our regression analysis, we assume that participants with missing data were coded as “No” for the missing APO variable. For multivariable analysis, variables were selected a priori based on their association with adverse pregnancy outcomes in the literature [17–19]. Covariates included age, income, education, depression, stress level, age of menarche, elevated BMI, and hypertension. All data processing and evaluations were executed using R statistical software (version 4.2.3).

### Ethics

This is a sub-study of the Haiti CVD Cohort Study, approved by the institutional review boards of Weill Cornell Medicine and GHESKIO (WCM 1803019037-27). All participants provided informed consent for the use of their information in cardiovascular disease (CVD) research. We follow the Declaration of Helsinki and The Belmont Report for the Protection of Human Subjects of Biomedical and Behavioral Research.

## Results

### Sociodemographic and clinical characteristics

Among 1,163 women reporting reproductive health data, the median age was 43 years, ranging from 18 to 84 years (Table 1). Supplement Table 1 reports characteristics between women with and without reproductive health data. Notably, women who were unable to complete the survey were, on average, younger (median age 38 years, IQR 26–54), more likely to have secondary or higher education (63.0%), and had lower median BMI (24.8 kg/m², IQR 21.6–29.7) as well as lower stress scores (median 8.0, IQR 6.0–10.0). The majority of women with reproductive health data reported not being married (61.0%, *n* = 708), being unemployed (67.0%, *n* = 771), and living on less than 1 USD daily (68.0%, *n* = 791). 61% (61.0%, *n* = 706) were overweight or obese, with Body Mass Index (BMI) > 25 kg/m2. One-third of the participants (*n* = 392) had hypertension. The women reported an average perceived stress level in the moderate to high range, respectively (69.0%, *n* = 804) (18.0%, *n* = 206); 31.0% (*n* = 364) reported mild depression, and 17.0% (*n* = 195) moderate to severe depression levels.

**Table 1:** Enrollment characteristics of women reporting reproductive health data (*n* = 1,163)

Characteristics of women with reproductive health data	Participants *N*:1163
Age, Median (IQR; Range)	43 (31, 55; 18–84)
18–29	256 (22.0%)
30–39	236 (20.0%)
40–49	235 (20.0%)
50–59	238 (20.0%)
> 60	197 (17.0%)
Missing data	< 1%
Marital status	
Married	451 (39.0%)
Non married	708 (61.0%)
Missing data	< 1%
Education level	
Primary or lower	530 (46.0%)
Secondary or higher	629 (54.0%)
Missing data	< 1%
Income	
< 1 USD per day	791 (68.0%)
1–10 USD per day	144 (12.0%)
> 10 USD/day	224 (19.0%)
Missing data	< 1%
Employment status	
Employed	60 (5.2%)
Homemaker	6 (0.5%)
Merchant	316 (27.0%)
Retried	1 (< 0.1%)
Student	5 (0.4%)
Unemployed	771 (67.0%)
Missing data	< 1%
BMI, kg/m²: Median (IQR)	26.6 (22.8, 30.6)
Underweight < 18.5	58 (5.0%)
Normal weight (18.5–25)	398 (34.0%)
Overweight (25–30)	380 (33.0%)
Obese > 30	326 (28.0%)
Missing data	< 1%
Hypertension	
Hypertension	
Missing data	< 1%
Perceived Stress Score: Median (IQR)	8.00 (7.0, 10.0)
Low (< 6)	149 (13.0%)
Moderate (6–10)	804 (69.0%)
High (≥ 11)	206 (18.0%)
Missing data	< 1%
Depression (PHQ-9 score):	
None (< 6)	600 (52.0%)
Mild (6–10)	364 (31.0%)
Moderate to Severe (≥ 11)	195 (17.0%)
Missing data	< 1%

### Reproductive health characteristics

The median age at menarche was 14 years (IQR, 12–16 years; range, 8–22 years) (Table 2). The median age at menopause was 48 years (IQR, 44–50; range, 30–63) (Table 2). Notably, there is a long-term decrease in the median age at menarche when comparing older birth cohorts with younger ones (Fig. 1). Overall, for women born between 1930 and 1970, the average age at menarche was 15 years; for women born between 1970 and 1990, it was 14 years; and subsequently, it decreased to 13 years from 1990 to 2012 (*p* < 0.001) using the Kruskal-Wallis rank sum test. The reported age of menopause from 1930 to 1950 is 47 (IQR 43–50), 1950–1970 is 49 years (IQR 45–52), and 1970–1990 is 46 years (IQR 42–49) (*p* < 0.001).

**Table 2:** Reproductive health profile of women reporting reproductive health data (*n* = 1,163)

Reproductive Health Characteristics	*N*: 1163
Age (years) at Menarche, Median (IQR; Range)	14.00 (12.00, 16.00; 8–22)
Less than 12 years (early)	87 (7.5%)
12–16 years	897 (77.0%)
Above 16 years (late)	177 (15.0%)
Missing data	< 1.0%
Infertility	
History of infertility	130 (11.0%)
Missing data	< 1.0%
Menstrual cycle	
Regular	687 (59.0%)
Irregular	476 (41.0%)
Parity	
0	156 (13.0%)
1–2	321 (28.0%)
3–5	456 (39.0%)
6 +	230 (20.0%)
Pregnancy-related characteristics	women with 1 or more pregnancies (*n* = 1007)
History of pregnancy loss	515 (51.0%)
Missing data	17 (1.7%)
History of preterm births	132 (13.0%)
History of pregnancy complications	244 (24.0%)
Missing data	3 (< 1%)
History of breastfeeding	883 (88.0%)
Missing data	2 (< 1.0%)
Age of menopause (IQR; Range)	48.0 (44.0, 50.0; 30–63) N:540
30–39 Y (premature%)	40 (7.4%)
40–45 Y (early%)	163 (30.0%)
46–54 Y	317 (59.0%)
> 55 Y (late%)	20 (3.7%)
Missing data	< 1.0%

*Pregnancy complications include a history of high blood pressure, diabetes in pregnancy, early delivery, and small size for gestational age

*For menopause, values under 30 were removed

A total of 41.0% (*n* = 476) of participants reported menstrual irregularity. A history of infertility was reported among 11.0% (*n* = 130) of our cohort. Among 1,163 women, 1,007 (86.5%) had a history of parity. Grand multiparity—defined as six or more births—was reported by 20.0% (*n* = 230) of the participants. 88.0% (*n* = 883) of women reported breastfeeding.

**Fig. 2:**
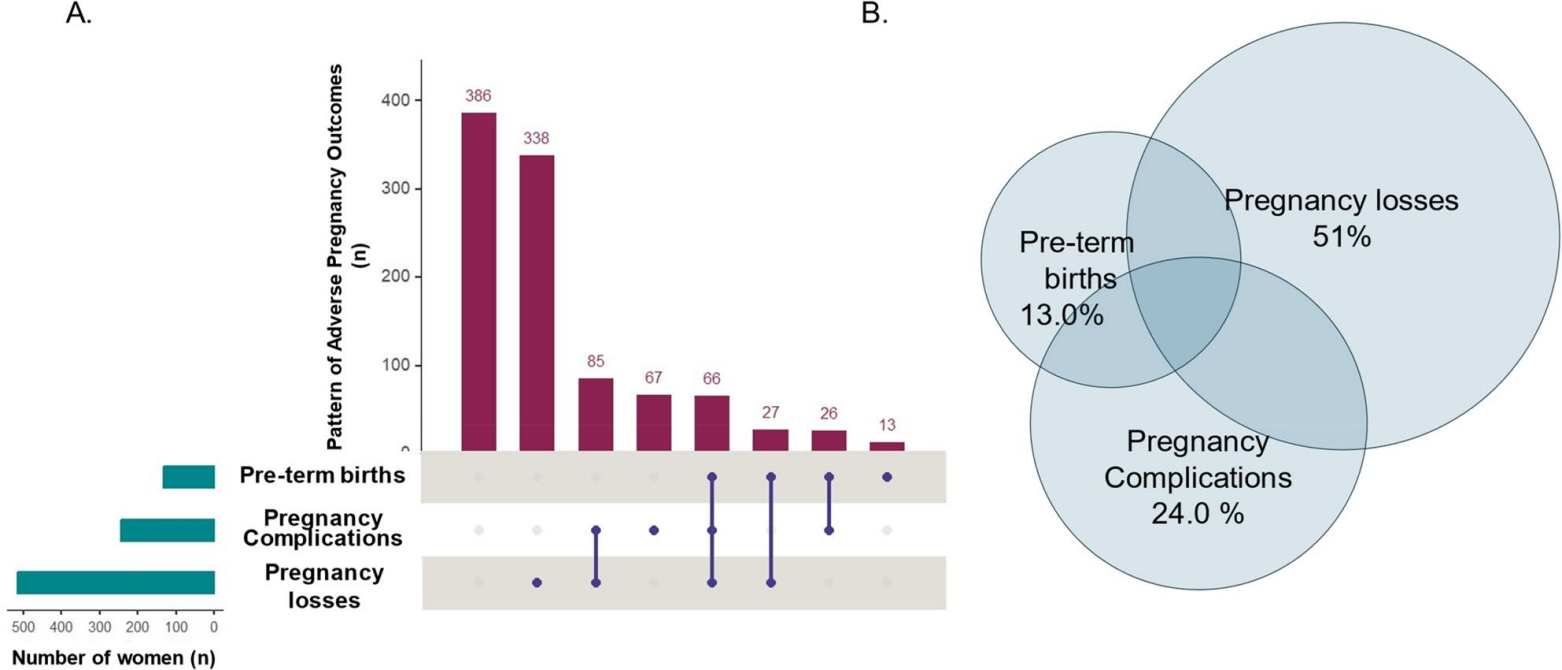
Adverse Pregnancy Outcomes: Individual burden and co-occurrence of key adverse pregnancy outcomes. **A** Counts of women with one or more reported adverse pregnancy outcomes. **B** Visual summary of co-occurrence of adverse pregnancy outcomes n: 623

Among 1007 women with *≥* one pregnancy, 61.7% (*n* = 623) experienced at least one APO, with 13.8% (*n* = 139) experiencing more than one and 6.5% (*n* = 67) experiencing three (Fig. 2). Pregnancy loss was the most frequent, with 51.0% (*n* = 515) of women, followed by 24.0% (*n* = 244) of women with pregnancy complications, which include gestational hypertension, pre-eclampsia, gestational diabetes, and intrauterine growth restriction, and 13.0% (*n* = 132) with a history of preterm births (Table 2). The most common combination of different APO was a history of pregnancy loss together with pregnancy complications (, which include hypertensive disorders of pregnancy, gestational diabetes, and small-for-gestational-age infants The most common combination of different APO were history of pregnancy loss and pregnancy complications, defined as hypertensive disorders of pregnancy, gestational diabetes, and small size for gestational age (24.0%, *n* = 86).

Women who reported APO were more likely to have hypertension (PR: 1.14, 95%CI: 1.02, 1.28). There is also a significant association between a history of APO and higher BMI, notably with both overweight (PR: 1.17, 95%CI: 1.04, 1.32) and obese categories (PR 1.16, 95%CI: 1.03–1.32) compared to normal/underweight BMI. Early or late age of menarche was not significantly associated with women experiencing APO, with a PR of 1.02 (CI 0.90, 1.15, *p* = 0.804) and a PR of 1.00 (CI 0.89, 1.12, *p* = 0.946), respectively. Other factors associated with reported APO (Table 3) include participants who report having a secondary or higher level of education, PR 1.28 (CI 1.14, 1.43, *p* < 0.001). A reported history of moderate perceived stress was also found to be associated with APO, PR 1.19 (CI 1.00, 1.42, *p* < 0.05). Baseline income and severity of depression did not have a significant association with a history of APO.

**Table 3:** Factors associated with adverse pregnancy outcomes

	N	Univariable(PR, 95% CI)	p-value	Multivariable (PR, 95% CI)	p-value
Age					
< 40 years	367	*Ref.*		*Ref.*	
≥ 40 years	639	0.92 (0.84, 1.02)	0.106	0.98 (0.87, 1.10)	0.681
Income					
< 1 USD/day	667	*Ref.*		*Ref.*	
≥ 1 USD/day	336	1.02 (0.92, 1.13)	0.750	0.99 (0.89, 1.10)	0.847
Education					
Primary or less	512	*Ref.*		*Ref.*	
Secondary or higher	491	**1.23 (1.11, 1.36)**	**<0.001**	**1.28 (1.14, 1.43)**	**<0.001**
Depression (PHQ-9)					
None to Mild (<10)	167	*Ref.*		*Ref.*	
Moderate to Severe (11+−20)	836	1.01 (0.89, 1.16)	0.824	1.01 (0.88, 1.16)	0.882
Perceived Stress Score					
Low (<6)	116	*Ref.*		*Ref.*	
Moderate (6–10)	706	1.17 (0.98, 1.40)	0.084	1.19 (1.00, 1.42)	0.051
High (≥ 11)	181	1.17 (0.95, 1.43)	0.136	1.18 (0.96, 1.44)	0.112
Age at menarche					
13–14 years	332	*Ref.*		*Ref.*	
≤ 12 years (early)	258	1.03 (0.91, 1.16)	0.689	1.02 (0.90, 1.15)	0.804
>15 years (late)	416	0.96 (0.85, 1.07)	0.446	1.00 (0.89, 1.12)	0.946
BMI					
Underweight/Normal (BMI < 24.9)	351	*Ref.*		*Ref.*	
Overweight (BMI 25.0–29.9.0.9)	349	**1.17 (1.04, 1.32)**	**<0.05**	**1.15 (1.02, 1.30)**	**<0.05**
Obese (BMI≥ 30.0)	306	**1.16 (1.03, 1.32)**	**<0.05**	**1.15 (1.01, 1.30)**	**<0.05**
Hypertension	373	1.06 (0.96, 1.18)	0.216	**1.14 (1.02, 1.28)**	**<0.05**

*Using log-binomial regression to calculate prevalence ratio

N= 1007 APO = 623 No APO = 387

Items in bold have a significant association

## Discussion

Our study found that the most common adverse reproductive health factors in this cohort of urban women living in extreme poverty were high rates of adverse pregnancy outcomes (APO) and a trend of earlier menarche over time across successive birth cohorts. Among APO, the most commonly reported were pregnancy losses and pregnancy complications. A reported history of APO was associated with being overweight and obese, as well as with hypertension. 

Adverse reproductive health characteristics negatively affect women’s health outcomes in both the short term and long term, impacting maternal health and raising the risk of chronic diseases throughout a woman’s lifespan [6]. Reproductive hormone variations, primarily estrogens, androgens, and progestins, impact disease pathogenesis by modifying signaling pathways mediated by sex steroid receptors in various organs [20]. Consequently, women’s reproductive lifespan, from menarche and parity to menopause, is associated with various chronic diseases like metabolic syndrome [21], cardiovascular disease [22–24], autoimmune diseases [20], infectious diseases [25], and malignancies [26], including breast cancer [27]. Globally, the burden of disease among women has shifted significantly towards chronic diseases, including cardiometabolic diseases, cancers, depression, and dementia, with poor outcomes particularly in low-income countries like Haiti [28]. Adverse reproductive health characteristics may represent a first warning that a woman is vulnerable to future chronic diseases [6, 29]. In Haiti, cardiovascular disease has become the leading cause of death, with an estimated 21.3% of adult deaths [30], and 24.4% of women’s deaths [30]. Identifying the relationship between adverse reproductive health outcomes and chronic disease, particularly heart disease, is essential, as these outcomes may serve as early indicators to help target women at risk for timely screening, prevention, and treatment.

In this study, we found that 61.7% (*n* = 623) of women reported an adverse pregnancy event, and approximately 20.3% (*n* = 206) reported two or more adverse reproductive health outcomes. Our prevalence of adverse pregnancy outcomes was over two-fold higher when compared to data in the US and other low- and middle-income countries (LMICs). While there is heterogeneity in defining adverse pregnancy outcomes, data from the National Nurses’ Health Study in the US estimate that 29% of parous study participants have had one adverse pregnancy outcome defined as low birth weight, fetal growth restriction, preterm delivery, hypertensive disorders of pregnancy, and gestational diabetes mellitus (GDM) [31]. Our findings are also significantly higher than what has been described in Ghana (prevalence of 19.0- 44.6%) [32, 33], Uganda (37.0%) [33], and a pooled prevalence of adverse birth outcomes of 29.7% from a study using the most recent Demographic and Health Surveys (DHS) to survey ten Sub-Saharan African countries [34]. The explanations for our findings are likely multifactorial, including medical and obstetric conditions, socioeconomic factors, environmental factors, and country-specific health system failures [35]. Women from LMICs, like Haiti, bear a high burden of infectious and obstetric diseases, which can lead to APO [36–38]. A crucial aspect may be related to the degree of poverty that women in urban Haiti face, which is devastatingly high and affects already fragile health care services, ability to access health care, quality of health care, and individual-level social determinants of health. Studies from Ireland and the UK show a higher rate of adverse pregnancy outcomes, particularly stillbirths, for women of low socioeconomic background [39]. High parity and a short interval between births are also associated with APO, and 20% (*n* = 230) of our cohort reported grand multiparity, with six or more pregnancies [40]. Environmental factors, such as toxic heavy metals including lead and cadmium, are associated with a range of APO [41, 42]. Lead exposure is associated with preterm births and pre-eclampsia [43, 44]. We previously demonstrated that blood lead levels in Haiti are up to five times higher than in the US, so it remains to be investigated whether heavy metals are a significant factor influencing reproductive outcomes in our cohort [42]. Further research is needed to improve the understanding of the interplay between poverty at the individual, clinic, and health system levels and adverse reproductive health outcomes like APO in LMICs.

The most common adverse reproductive health event reported was pregnancy loss, with 54.0% (*n* = 337) of women having had a stillbirth or a miscarriage. We were unable to ascertain gestational age at the time of loss to distinguish between stillbirths and miscarriages. Stillbirth is defined as the delivery of a fetus that has died following at least 22 weeks of gestation [45]. Stillbirth is recognized to be a silent epidemic and contributes significantly to worldwide neonatal mortality, with 84% of the 2 million stillbirths globally occurring in LMICs [46, 47]. Risk factors for stillbirths include maternal infections like HIV, hepatitis, and other factors such as obesity, nulliparity, chronic hypertension, diabetes, smoking, alcohol use, and social determinants of health, spanning poverty, poor nutrition, and lack of access to clinic-based care [46, 48, 49]. Women with prior stillbirths are at higher risk of having other complications in subsequent pregnancies, including gestational diabetes and hypertensive disorders of pregnancy [50]. Miscarriage refers to pregnancy loss before viability, 22 weeks [51]. The pooled data from multiple studies estimate the average population prevalence of at least one miscarriage to be approximately 10.8% [52]. Risk factors for miscarriages include pre-pregnancy factors such as age above 30, prior miscarriages, underweight, and obesity, and factors during pregnancy like alcohol consumption [53]. Similarly to stillbirths, a history of miscarriage is associated with other APO [52]. Our findings support this conclusion, as many participants with a history of pregnancy loss also reported experiencing preterm births (*n* = 27) and other pregnancy complications (*n* = 86), including hypertensive disorders, gestational diabetes, and being small for gestational age. Surprisingly, women with higher levels of education were associated with an increased risk of APO. Reasons for this finding could be that more educated women have greater access to perinatal clinical care and thus were diagnosed and educated about their adverse pregnancy events, such as being told by a provider that they had a stillbirth or preterm birth, as compared to less educated women. Similarly, women with lower educational attainment may underreport their APO as they may be generally less aware of what defines an APO or have less interaction with the formal healthcare system where APO are diagnosed. Additionally, education may be correlated with unmeasured confounders, such as our participants’ social and environmental characteristics, which can also impact APO. Additional qualitative data is needed to unpack this finding.

We found that both high BMI and hypertension were significantly associated with adverse pregnancy outcomes in the cohort. Prior literature has found that women with a pre-pregnancy BMI >25 or higher are at higher risk of miscarriage and stillbirth [54]. There is a high rate of obesity in the cohort (61.0%, *n* = 706) despite devastating poverty and food insecurity, which could be contributing to the high prevalence of reported APO. More research is necessary to understand obesity-related adverse pregnancy outcomes and explore effective obesity treatments in low-income countries, considering the rising prevalence of obesity. Hypertension was significantly linked with APO. Hypertension is the predominant modifiable cardiovascular risk factor for heart disease among women in LMICs, including Haiti [55, 56]. A total of 16.0% of maternal mortality in LMICs has been attributed to hypertensive disorders of pregnancy [57]. The risk of developing cardiovascular disease after a pregnancy with hypertensive disorders is attributed to persistent hypertension [58, 59]. Further studies are necessary to establish a causal relationship between elevated blood pressure and adverse pregnancy outcomes in our cohort, and vice versa. However, the abundance of literature on high rates of hypertension disorders of pregnancy in LMICs underscores the urgent need for implementation strategies for evidence-based screening, prevention, and treatment interventions in these settings.

We also report a decreasing age of menarche over time in Haiti. We found the median age of menarche to be 14, with the older cohorts closer to 15 and a decline among the younger group to approximately 13 years. Similarly, Barnes-Josiah et al. investigated trends in the age at menarche among 10,563 pregnant Haitian women as part of a longitudinal study on characteristics of maternal mortality in 1995 and noted a generational decline in the reported mean age [60, 61]. This aligns with a global trend of a declining average age of menarche in LMICs, from 14.6 years in the 1930 s to 12.8 years in the 2000 s cohorts, potentially at a faster rate than in high-income countries [62, 63]. 7.5% (*n* = 87) of our cohort had an age of menarche before 12 years. Factors that contribute to early menarche have primarily been studied in high-income countries, but include higher childhood BMI and adiposity attributed to the impact of leptin and insulin-like growth factor on the hypothalamic-pituitary-gonadal axis [64, 65]. Other factors, such as prenatal exposures like maternal smoking and maternal diseases such as diabetes, have been linked to an earlier trend of menarche, but these exposures are relatively low in our cohort [66]. Psychosocial stressors such as childhood adversity have been implicated in early pubertal timing and may be particularly relevant for our cohort, given the level of poverty described, and should be further explored in subsequent studies [67–69]. Early menarche has been identified as a risk factor for reproductive cancers, including breast cancer and cardiovascular disease [70–73]. Furthermore, early menarche is linked to a greater incidence of diabetes, hypertension, and metabolic dysfunction [74–76]. Other adverse associations include a higher rate of depression and anxiety amongst girls who underwent early menarche. These implications are especially significant considering the potential strain it may place on an already overburdened healthcare system.

Our cohort’s reported median age at menopause was 48 years, with approximately 37.4% (*n* = 203) experiencing menopause before the age of 45, in contrast to the mean age of menopause of 50–51 years in high-income countries like the United States [77]. Early menopause is defined as menopause between 40 and 45 years, and premature ovarian insufficiency is before 40 years [78]. It has been reported that premature ovarian insufficiency and early menopause are more prevalent in lower-income countries at 3.7% and 12.2%, respectively [79]. The reason for the variations has not been well understood, and studies to date have yielded conflicting results regarding the impact of various risk factors, including socioeconomic status (such as income, education, and neighborhood) and other lifestyle factors (like physical activity and BMI) [80–82]. Early menopause is linked to chronic diseases like osteoporosis, cardiovascular disease, and mental health issues such as strokes, which are increasingly more prevalent in low- and middle-income countries [77].

Strengths of this study include a population-based cohort of women living in extreme poverty, including those from slum neighborhoods, who are not routinely included in population health surveys on reproductive health factors, as well as the number of reproductive health data points captured. The most recent comprehensive data on reproductive health in Haiti comes from the Demographic and Health Survey (EMMUS-VI 2016–2017) [51], which included only family planning and maternal care variables. Other notable strengths include that all participants identify as black Haitian, and black women have historically been underrepresented in clinical reproductive health research [83].

Limitations include that reproductive health factors were self-reported, which may lead to both recall and reporting biases; however, this approach is similar to that used in US cohorts, including NHANES [14], and prior research supports the validity and reliability of self-reported reproductive health characteristics [84–87]. There is also a potential for survivor bias in our analysis because we only included women who are alive, which may have excluded women who experienced severe complications related to APO. This could lead to an underestimation of the true burden of APO. Women who did not complete the survey tended to be younger, more educated, and had lower BMI and stress scores, which may limit how broadly our findings apply. Another limitation is our inability to distinguish between stillbirth and miscarriage when describing pregnancy loss, and to differentiate the individual components that make up APO. This lack of precision may obscure meaningful associations associated with each of these distinct adverse pregnancy outcomes. Our question on menstrual irregularities may not fully capture the spectrum of menstrual disorders. Additionally, reported infertility did not include the time frame defined by the FIGO algorithm, which could lead to an overestimation of that burden. When describing early and premature menopause, we also could not differentiate whether the etiology is surgical or medical, which can have further health implications [88]. While most participants reported breastfeeding, we lack data on the duration and exclusivity (e.g., mixed feeding), which limits our ability to evaluate the impact of breastfeeding patterns [89]. We also lack data on the timing of pregnancies and adverse pregnancy events, which restricts our ability to determine directionality and understand whether variables such as hypertension and high BMI preceded or resulted from adverse reproductive outcomes. This study is cross-sectional and is not designed to establish causal relationships between factors and incident APO.

## Conclusions

In conclusion, women living in urban Port-au-Prince, Haiti, under extreme poverty report nearly two-fold higher rates of adverse pregnancy outcomes than other low-income countries. Pregnancy loss and pregnancy complications, such as hypertensive disorders of pregnancy, are the most common and are associated with hypertension and obesity. We also found a decrease in the age of menarche over time. These findings emphasize the need for interventions to improve women’s reproductive health, focusing on pregnancy. These adverse reproductive health factors also place these women at increased risk for chronic diseases across the lifespan. There is a need for longitudinal studies to examine the long-term consequences of APO, and other reproductive health factors, in Haiti and other low-income countries. Further research is needed to explore women’s experiences with APO and related complications to inform the development and implementation of culturally relevant strategies to reduce the risk of adverse reproductive health outcomes, and related long-term morbidity, in Haiti and similar low-resource settings.

## Supplementary Information

Supplementary Material 1.

## Data Availability

The datasets analyzed for this study are available upon request. Proposals should be sent to the principal investigator at mam9365@med.cornell.edu.

## Abbreviations

APO: Adverse Pregnancy Outcomes
CVD: Cardiovascular disease
GDM: Gestational Diabetes Mellitus
LMICs: Low-and Middle-Income Countries
DHS: Demographic and Health Surveys
