# Supplementary material for: Reproductive health characteristics among women living in severe poverty in urban Haiti
Source: Reprod Health. 2025 Nov 24;22:266. doi: 10.1186/s12978-025-02219-3 (PMC12751971; doi:10.1186/s12978-025-02219-3)
Supplement: Supplementary file 1 — Supplementary Material 1. [file 12978_2025_2219_MOESM1_ESM.docx]

**Supplementary Table 1: Enrollment characteristics of women participants in the cardiovascular disease cohort**

| **Characteristics of women within the cardiovascular disease cohort** | **Total participants N = 1,744** | **With Repro Data, n = 1,163** | **Without Repro Data, n = 583** | **Deaths, n = 46** | **p-value** |
| --- | --- | --- | --- | --- | --- |
| **Age, Median (IQR, Range)** | **42 (29, 55)** | **43 (31, 55)** | **38 (26, 54)** | **59 (42, 66)** | **<0.001** |
| 18-29 | 450 (26.0%) | 256 (22.0%) | 194 (33.0%) | 4 (8.7%) |  |
| 30-39 | 353 (20.0%) | 236 (20.0%) | 117 (20.0%) | 5 (11.0%) |  |
| 40-49 | 335 (19.0%) | 235 (20.0%) | 100 (17.0%) | 7 (15.0%) |  |
| 50-59 | 315 (18.0%) | 238 (20.0%) | 77 (13.0%) | 10 (22.0%) |  |
| 60+ | 291 (17.0%) | 196 (17.0%) | 95 (16.0%) | 20 (43.0%) |  |
| **Marital status** |  |  |  |  | **0.4** |
| Married | 661 (38.0%) | 451 (39.0%) | 210 (36.0%) | 16 (35.0%) |  |
| Non married | 957 (55.0%) | 626 (54.0%) | 331 (57.0%) | 23 (50.0%) |  |
| Widowed/Divorced/Separated | 118 (6.8%) | 81 (7.0%) | 37 (6.4%) | 7 (15.0%) |  |
| Missing data | <1 % | <1 % | <1 % | <1% |  |
| **Education level** |  |  |  |  | **<0.001** |
| Secondary or Higher | 993 (57.0%) | 629 (54.0%) | 364 (63.0%) | 19 (41.0%) |  |
| Primary or Lower | 743 (43.0%) | 529 (46.0%) | 214 (37.0%) | 27 (59.0%) |  |
| Missing data | <1 % | <1 % | <1 % | <1 % |  |
| **Income** |  |  |  |  | **0.2** |
| < 1 USD / day | 1,208 (70.0%) | 790 (68.0%) | 418 (72.0%) | 39 (85.0%) |  |
| 1 to 10 USD / day | 207 (12.0%) | 144 (12.0%) | 63 (11.0%) | 6 (13.0%) |  |
| > 10 USD / day | 321 (18.0%) | 224 (19.0%) | 97 (17.0%) | 1 (2.2%) |  |
| Missing data | <1 % | <1 % | <1 % | <1% |  |
| **Employment status** |  |  |  |  | **0.3** |
| Employed | 81 (4.7%) | 60 (5.2%) | 21 (3.6%) | 0 (0.0%) |  |
| Homemaker | 9 (0.5%) | 6 (0.5%) | 3 (0.5%) | 0 (0.0%) |  |
| Merchant | 455 (26.0%) | 316 (27.0%) | 139 (24.0%) | 8 (17.0%) |  |
| Retired | 2 (0.1%) | 1 (<0.1%) | 1 (0.2%) | 0 (0.0%) |  |
| Student | 8 (0.5%) | 5 (0.4%) | 3 (0.5%) | 0 (0.0%) |  |
| Unemployed | 1,180 (68.0%) | 770 (66.0%) | 410 (71.0%) | 38 (83.0%) |  |
| Missing data | <1 % | <1 % | <1 % | <1 % |  |
| **BMI, kg/m²: Median (IQR)** | **26.1 (22.2, 30.3)** | **26.6 (22.8, 30.6)** | **24.8 (21.6, 29.7)** | **25.9 (21.3, 30.9)** | **<0.001** |
| Underweight <18.5 | 93 (5.3%) | 58 (5.0%) | 35 (6.0%) | 17 (37.0%) |  |
| Normal weight (18.5-25) | 658 (38.0%) | 397 (34.0%) | 261 (45.0%) | 12 (26.0%) |  |
| Overweight (25-30) | 532 (31.0%) | 380 (33.0%) | 152 (26.0%) | 13 (28.0%) |  |
| Obese | 460 (26.0%) | 326 (28.0%) | 134 (23.0%) | 4 (8.7) |  |
| Missing data | <1 % | <1 % | <1 % | <1 % |  |
| **Perceived Stress Score: Median (IQR)** | **8.00 (7.00, 10.00)** | **8.00 (7.00, 10.00)** | **8.00 (6.00, 10.00)** | **9.00 (8.00, 10.00)** | **0.002** |
| Low (< 6) | 250 (14.0%) | 149 (13.0%) | 101 (17.0%) | 4 (8.7 %) |  |
| Moderate (6-10) | 1,201 (69.0%) | 804 (69.0%) | 397 (69.0%) | 31 (67.0%) |  |
| High (≥ 11) | 285 (16.0%) | 205 (18.0%) | 80 (14.0%) | 11 (24.0%) |  |
| Missing data | <1 % | <1 % | <1 % | <1 % |  |
| **Depression (PHQ-9 score):** | **5.0 (2.0, 9.0)** | **5.0 (2.0, 9.0)** | **5.0 (3.0, 9.0)** | **5.5 (4.0, 11.0)** | **0.5** |
| None (< 6) | 906 (52.0%) | 600 (52.0%) | 306 (53.0%) | 23 (50.0%) |  |
| Mild (6-10) | 533 (31.0%) | 363 (31.0%) | 170 (29.0%) | 11 (24.0%) |  |
| Moderate to Severe (≥11) | 297 (17.0) | 195 (17.1) | 102 (17.0) | 12 (26.0%) |  |
| Missing data | <1 % | <1 % | <1 % | <1 % |  |
| **Hypertension** | **575 (33.0%)** | **392 (34.0%)** | **183 (31.0%)** |  | **0.3** |
| *Repro: reproductive | | | | | |

**Supplementary Table 2: Reproductive health questionnaire completed by women participants in the cardiovascular disease cohort**

| **Reproductive Health Questionnaire, English version** |
| --- |
| - What age were you when you had your first period? |
| - Do you have a history of or currently have irregular periods, ie, the time between your periods changes a lot, or the length of time you have a period changes a lot? |
| - How many total pregnancies have you had? |
| - How many children were born alive? |
| - Full term births. |
| - Preterm births (prior to 37 weeks gestation). |
| - Abortions (including miscarriages) |
| - Have you had difficulty getting pregnant in the past or now |
| - Have you had any complications during your pregnancies (high blood pressure, diabetes, early delivery, small baby)? |
| - Did you breastfeed any of your children? |
| - What age were you when you had menopause, ie your period stopped? |
| **Reproductive Health Questionnaire, Creole version** |
| - Ki laj ou te genyen lè ou te fòme (gen règ ou)? |
| - Eske ou konn gen règ iregilye, sa vle di li pa vini nan tan li dwe vini, li varye? |
| - Èske w te gen difikilte pou w vinn ansent? |
| - Konbyen fwa ou te ansent. |
| - Konbyen timoun ou akouche vivan? |
| - Konbyen timoun ou akouche sou nèf mwa? |
| - Konbyen timoun ou te akouche avan lè (avan 9 mwa) |
| - Konbyen fwa ou te jete timoun oubyen pèdi timoun lan avan ou akouche (foskouch) |
| - Èske w te gen nenpòt konplikasyon pandan gwosès ou (tansyon wo, dyabèt, akouchman bonè, bebe ki fèt piti anpil)? |
| - Èske ou te bay tete? |
| - Ki laj ou te genyen lè ou te fè menopoz ou, sa vle di règ ou te kanpe? |

**Supplementary Table 3: Operational Definitions of Key Reproductive Variables**

| **Variable** | **Definition** |
| --- | --- |
| **Early Menarche** | Onset of menstruation before age 12 |
| **Late Menarche** | Onset of menstruation after age 15 |
| **Pregnancy loss** | The difference between total pregnancies and live births. It is defined as present if ≥0 and includes stillbirths and miscarriages. |
| **Pregnancy complications** | Includes all the following:  – Hypertensive disorders of pregnancy  – Gestational diabetes  – Small-for-gestational-age birth |
| **Adverse Pregnancy Outcomes (APOs)** | Includes any of the following:  - History of pregnancy loss  - Preterm birth  - Pregnancy complications: |
| **Premature menopause** | Menopause before age 40 |
| **Early menopause** | Menopause before age 45 |

**Supplementary Figure 1.**

**Haiti CVD Cohort Female Participants**

(n = 1746; 100%)

**Exclusion** (n =577)

- Deaths before completing survey (n=46, 2.6%)
- Study exit (n=10, 0.5%)
- Missing reproductive health questionnaire (n= 527, 30,0%)

**Completed reproductive health questionnaire**

(n = 1163; 66.6%)

# 
